# A Review of Bacteriochlorophyllides: Chemical Structures and Applications

**DOI:** 10.3390/molecules26051293

**Published:** 2021-02-27

**Authors:** Chih-Hui Yang, Keng-Shiang Huang, Yi-Ting Wang, Jei-Fu Shaw

**Affiliations:** 1 Department of Biological Science and Technology, College of Medicine, I-Shou University, No.8, Yida Rd., Jiaosu Village, Yanchao District, Kaohsiung City 82445, Taiwan; chyang@isu.edu.tw; 2 Pharmacy Department of E-Da Hospital, Kaohsiung City 82445, Taiwan; 3 Taiwan Instrument Research Institute, National Applied Research Laboratories, Taipei City 106214, Taiwan; 4 The School of Chinese Medicine for Post-Baccalaureate, I-Shou University, Kaohsiung City 82445, Taiwan; huangks@isu.edu.tw

**Keywords:** bacteriochlorophyllides, bacterochlorophylls, photosensitizers, immunosensors, dye-sensitized solar cell

## Abstract

Generally, bacteriochlorophyllides were responsible for the photosynthesis in bacteria. Seven types of bacteriochlorophyllides have been disclosed. Bacteriochlorophyllides *a*/*b*/*g* could be synthesized from divinyl chlorophyllide *a*. The other bacteriochlorophyllides *c*/*d*/*e*/*f* could be synthesized from chlorophyllide *a*. The chemical structure and synthetic route of bacteriochlorophyllides were summarized in this review. Furthermore, the potential applications of bacteriochlorophyllides in photosensitizers, immunosensors, influence on bacteriochlorophyll aggregation, dye-sensitized solar cell, heme synthesis and for light energy harvesting simulation were discussed.

## 1. Introduction

**Figure 1:**
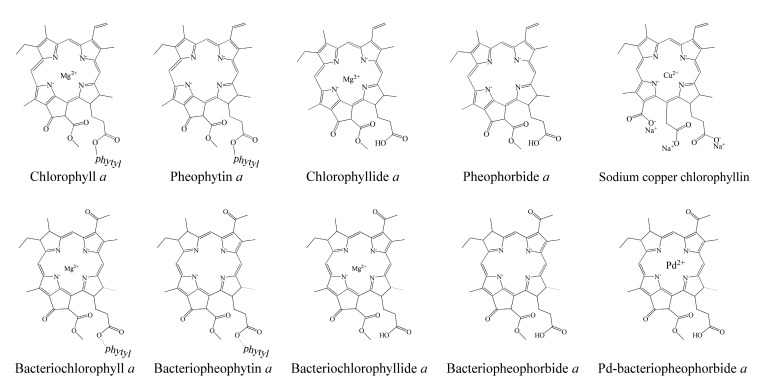
The compounds relative to bacteriochlorophyllides.

Bacteriochlorophyllides are involved in photosynthesis without production of oxygen in bacteria. Chlorophototrophic bacteria employ chlorophylls or bacteriochlorophylls to capture light as the source of energy for chemical synthesis [1]. The similar structures or relative compounds to bacteriochlorophyllides are shown in Figure 1. The structure features of bacteriochlorophyllide *a* were with tetrapyrroles, a dicarboxylic acid, a methyl ester, a methyl ketone and magnesium ion. Among them, tetrapyrroles are one of the macrocyclic compounds which existed in nature and played an important role in living organisms [2,3,4,5]. Previous study exhibited that bacteriochlorophyllides were found and accumulated in mutant strains of *Rhodopseudomonas spheroids* [6]. Bacteriochlorophyllides are the esterifying alcohol-free form of bacteriochlorophyll. In 1954, the preparation and conversion of bacteriochlorophyll into bacteriochlorophyllide in methanol by chlorophyllase was firstly described [7].

### 1.1. A Brief History of Bacteriochlorophylls

**Figure 2:**
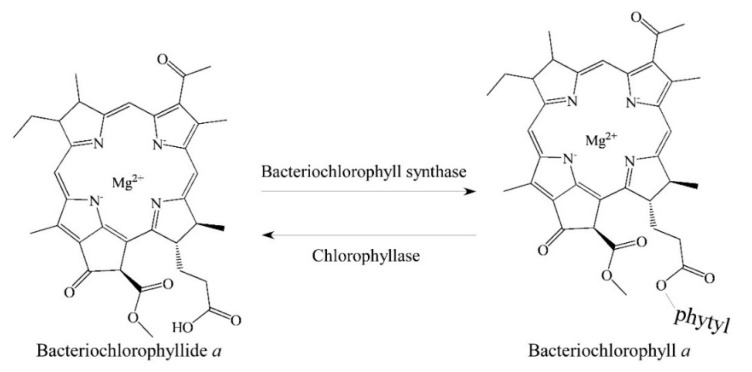
The relation between bacteriochlorophyllide *a* and bacteriochlorophyll *a*.

It is well known that the hydrolysis of bacteriochlorophyllase could remove a long alkyl chain (e.g., phytyl group) from bacteriochlorophyll *a* to generate bacteriochlorophyllide *a* [8,9,10] (Figure 2). For better understanding the precursor of bacteriochlorophyllides, a discovery history of bacteriochlorophylls was descripted. Generally, bacteriochlorophylls *a* to *f* were named by the order of discovery. The first isolation of bacteriochlorophyll *a* was reported in 1935 by Hans Fischer and Johann Hasenkamp [11]. Later, they also proposed the chemical structure and formulation of bacteriochlorophyll *a* in 1937 [12]. The discovery of bacteriochlorophyll *b* was isolated in 1963 from *Rhodopseudomonas* sp. photosynthetic bacterium by paper chromatography [13]. The bacteriochlorophylls *c* and *d* originally were called the *Chlorobium* chlorophyll-660 and -650, respectively [14]. Bacteriochlorophyll *e* was found and isolated from *Chlorobium phaeobacteroides* and *Chlorobium phaeovibrioides* by thin-layer chromatography in 1975 [15,16]. Then, the nomenclature of the bacteriochlorophylls *c*, *d* and *e* were further clarified in 1994 [17]. Although, the name bacteriochlorophyll *f* was proposed in 1975 due to the similarity with bacteriochlorophyll *e* [15]. The existence of bacteriochlorophyll *f* in nature from *Chlorobaculum limnaeum* was reported until 2012 [18,19,20]. Bacteriochlorophyll *g* was firstly found in *Heliobacterium chlorum* (heliobacteria) [21,22].

### 1.2. The Chemical Structure of Bacteriochlorophyllides

Seven types of bacteriochlorophyllides were found from different bacteria [1]. For example, bacteriochlorophyllides *a*/*b*/*g* which have bacteriochlorin ring structure were found in the purple phototrophic bacteria [23,24,25]. In addition, bacteriochlorophyllides *c*/*d*/*e*/*f* which have chlorin ring structure were found in green bacteria [2,26,27,28,29]. The unique structural features allow bacteriochlorophyllide *c*/*d*/*e*/*f* to form the supramolecular structures and self-assemble within chlorosomes.

The molecular formula, molecular weight and structure of bacteriochlorophyllides are summarized in Table 1. Bacteriochlorophyllide *a* (C_35_H_36_MgN_4_O_6_) was the most abundant bacteriochlorin. It was found in green bacteria, most purple bacteria and *Gemmatimonas phototrophica* [1,30]. Bacteriochlorophyllide *a* possessed a bacteriochlorin ring and an acetyl group in C3 [6,31]. Bacteriochlorophyllide *b* (C_35_H_34_MgN_4_O_6_) possessed a bacteriochlorin ring and an ethylidene group at C8 [10]. Bacteriochlorophyllide *c* (C_31_H_28_MgN_4_O_4_(R_1_)(R_2_)) was different from bacteriochlorophyllide *a* in a *α*-hydroxyethyl group at C3, R_1_ group at C8, R_2_ group at C12, a hydrogen at C13^2^ and a methyl group at C20. The R_1_ group of bacteriochlorophyllide *c* could be isobutyl, *n*-propyl or ethyl group, while that of the R_2_ group could be ethyl or methyl group. Saga and Yamashita studied the esterifying alcohols to the precursor of bacteriochlorophyll *c* and named this precursor as bacteriochlorophyllide *c* in green sulfur bacteria [32]. Bacteriochlorophyllide *c* exhibited a major pigment involved in light-harvesting in the green sulfur bacterium *Chloroflexus aurantiacus* and *Chlorobaculum tepidum* [33,34]. Bacteriochlorophyllide *d* (C_33_H_30_MgN_4_O_3_(R_1_)(R_2_) was analogous to bacteriochlorophyllide *c*, except that the C20 has a methyl group [15]. Bacteriochlorophyllide *e* (C_31_H_26_MgN_4_O_5_(R_1_)(R_2_) was different from bacteriochlorophyllide *c* by the presence of a formyl group at the C7 position of the chlorin ring. Bacteriochlorophyllide *f* was different to bacteriochlorophyllide *e* at C20 without methyl group [35]. Bacteriochlorophyllide *g* (C_35_H_34_MgN_4_O_5_) was bacteriochlorins with C3-vinyl group and C8-ethylidene group [36].

## 2. Biosynthetic Routes of Bacteriochlorophyllides

**Figure 3:**
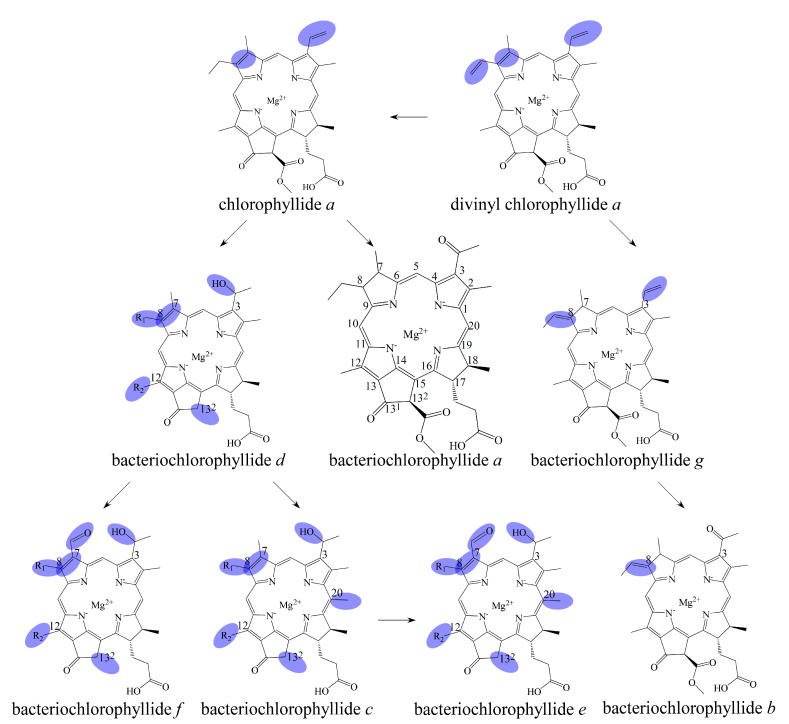
Biosynthetic routes among various bacteriochlorophyllides. The structure differences of bacteriochlorophyllide derivatives to bacteriochlorophyllide *a* were highlighted with spots.

The biosynthesis, regulation and functions progress of bacteriochlorophyllides have been disclosed [2,4,28,29]. The biosynthetic route of bacteriochlorophyllides was shown in Figure 3. Generally, the biosynthesis of bacteriochlorophyllides could be classified into two pathways; one of the pathways bring about bacteriochlorophyllides *b* and *g* and were derived from divinyl chlorophyllide *a*. The conversion of divinyl chlorophyllide *a* to bacteriochlorophyllide *g* proceeded via reduction of the double bond at C7 and C8. At the same time, this reduction generated a C8 ethlidene group in bacteriochlorophyllide *g*. For bacteriochlorophyllide *b* generation, two reactions were performed by C3^1^ hydratase and dehydrogenase from bacteriochlorophyllide *g*. The C3-vinyl side chain of bacteriochlorophyllide *g* was converted into a C3-acetyl group in bacteriochlorophyllide *b*.

Another pathway giving rise to the synthesis of bacteriochlorophyllides *a*, *c*, *d*, *e* or *f* was derivative from chlorophyllide *a*. Generally, chlorophyllide *a* could be converted into bacteriochlorophyllides *a* and *d*, and then bacteriochlorophyllide *d* could be employed to the synthesis of bacteriochlorophyllides *c*, *e* and *f*. The conversion of chlorophyllide *a* to bacteriochlorophyllide *a* proceeded via reduction, hydration and subsequent dehydrogenation, respectively. The reduction of the double bond occurred at C7 and C8 of chlorophyllide *a*. The hydration of the vinyl group proceeded to form a hydroxyethyl group at C3. The dehydrogenation of the hydroxyethyl group at C3 generated an acetyl group that resulted in the synthesis of bacteriochlorophyllide *a*.

For bacteriochlorophyllide *d* generation, demethoxycarbonylation, methylation and hydration of chlorophyllide *a* were occurred. The demethoxycarbonylation were occurred at C13^2^ to form a methylcarboxyl group. The C8 and C12 of chlorophyllide *a* was then carried on the methylation. Finally, the hydration acted at C3^1^ position, which produced a mixture of methylated species of bacteriochlorophyllide *d* [4]. Furthermore, bacteriochlorophyllide *d* could be the precursor for the synthesis of bacteriochlorophyllides *c*, *e* and *f*. For bacteriochlorophyllide *c* generation, bacteriochlorophyllide *d* could be converted into bacteriochlorophyllide *c* by a C20 methyltransferase [37,38]. For bacteriochlorophyllide *e* generation, C7 methyl group of bacteriochlorophyllide *c* was converted into a C7 formyl group by a radical S-adenosyl-l-methionine enzyme. As for bacteriochlorophyllide *f* generation, bacteriochlorophyllide *d* could be catalyzed by the same radical-SAM enzyme to form a formyl group at C7.

## 3. Applications

**Figure 4:**
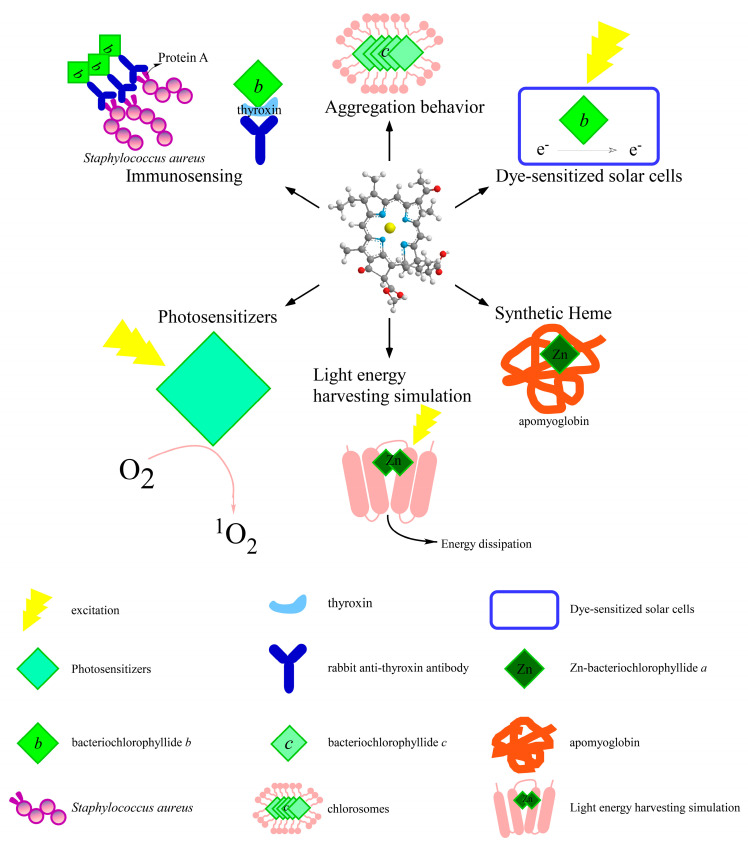
Some applications of bacteriochlorophyllides derivatives.

The attractive properties of bacteriochlorophyllides were having an intense absorption band in the long wavelength region, light sensitization, and photochemistry properties. Those properties are highly useful in various applications of bacteriochlorophyllides [39]. Herein, the applications of bacteriochlorophyllides were summarized and classified into six main types: photosensitizers, immunosensors, influence on bacteriochlorophylls aggregation, dye-sensitized solar cell, heme synthesis and light energy simulation (Figure 4).

### 3.1. For Photosensitizers Application

The favorable photophysical characters of bacteriochlorophyllides are their long-wavelength absorption, which comply with the specification of excellent photosensitizers in photodynamic therapy [39,40,41]. In recent years, there has been a growing interest in investigating of bacteriochlorophyllides derivatives with better photodynamic activity. However, bacteriochlorophyllides has instability problems that affect the extensive biomedical applications [27,42]. In order to stabilize bacteriochlorophyllides, many studies are devoted to the chemical modification or functional group substitutions of bacteriochlorophyllides. The functional properties and mechanism of those bacteriochlorophyll derivatives have been summarized and nicely discussed in several reviews and patents [39,40,41,43,44,45,46,47,48,49,50,51]. For example, Palladium-bacteriochlorophyllide *a* such as WST09 and WST11 (trade name TOOKAD^®^ and TOOKAD^®^ soluble) are excellent photosensitizers [47,52,53]. Noweski et al. used TOOKAD^®^ soluble WST11 and vascular-targeted photodynamic therapy to treat patients with localized prostate cancer within 3.5 years’ follow-up stage [54]. The treatment conditions were WST11 at 4 mg/kg, light energy at 200 J/cm, and a light density index ≥1. The overall successful focal ablation was 75% and 50% of patients were cancer-free in both the prostate lobes. Therefore, the authors summarized that this form of treatment may become a potential therapy in low-risk cancer. In addition, a new complex contained WST-11 and dextran (WST-D) was used to treatment of stiffening pathologically weakened corneas [55]. WST-D was illuminated with near infrared and used to treat rabbit corneas. The results indicated that the corneal thickness was increased at 1–4 days after treatment, and the epithelium was fully healed after 6–8 days. Therefore, WST-D combined with near infrared could safely stiffen the cornea. While with the research of natural-occurring bacteriochlorophyllides, the problem is the high cost of production. To gain access to the large amount of bacteriochlorophyllides, a green technology trend that uses enzyme to synthesis bacteriochlorophyllide was also reported [10]. A recombinant chlorophyllase named as CyanoCLH was cloned from a photosynthetic bacterium (*Cyanothece* sp. ATCC 51142) to produce bacteriochlorophyllide *a*. Then, bacteriochlorophyllide *a* could be converted into bacteriopheophorbide *a*, which was used to synthesize different types of photosensizers.

### 3.2. For Immunosensing Application

**Figure 5:**
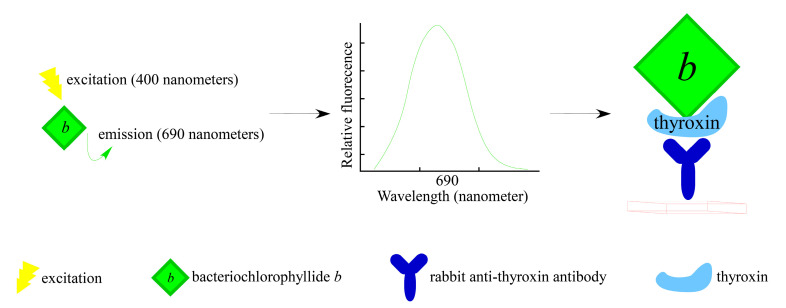
Schematic drawing of bacteriochlorophyllides used as immunosensors (Modified from Mahato et al. [56,58]).

Immunosensors are biosensing devices that include biorecognition element, transducers and the readout systems [56,57]. For immunosensing, bacteriochlorophyllides could be applied in immunoassays or specific targeting of bacteria. The luminous feature of bacteriochlorophyllide *b* was employed as a fluorescent labeled reagent in a fluora immunoassay system [58]. For example, bacteriochlorophyllide *b* was modified to form a complex with thyroxin, triiodothyronine or diogoxin (US4707454A) (Figure 5). Biorecognition was formed between thyroxin present in patient blood and rabbit anti-thyroxin serum pre-coated tubes. Bacteriochlorophyllide *b*- thyroxin as transducer was applied to convert observed signal into the quantifiable electrical signals. A spectrofluorometer was then used to measure the relative fluorescence for each sample (excitation: 400 nanometers; fluorescence emission: 690 nanometers). This assay detected the presence of the labeling agent comprising an excitation source illuminating a vessel with a photodetector directly within the illuminated area. Therefore, three advantages of bacteriochlorophyllide *b* as a fluorescent label agent (transducer) were used in an immunoassay: a relative high stoke shift, a longer fluoresce wavelengths and less expensive. For the specific targeting of bacteria, Scherz’s group investigated the phototoxicity of bacteriochlorophyllide-serine (Bchl-Ser) or bacteriochlorophyllide-Immunoglobulin G (Bchl-IgG) [59]. The conjugates were illuminated with light to determine the photodynamic efficiency. Results indicated that the Bchl-IgG were a highly specific bind to protein A on the wall of *Staphylococcus aureus*. The phototoxicity of Bchl-IgG conjugates was increased in a dose-dependent manner with LD_50_ = 1.7 μM. The LD_50_ of Bchl-Ser was 0.07 μM. The photocytotoxicities of Bchl-lgG and Bchl-Ser depend both on the concentration of sensitizer and light. However, the binding ability of Bchl-IgG was 66,000 Bchl-IgG/bacterium compared to that of Bchl-Ser, which was 1900,000/bacterium at LD_50_. A 29-fold higher photoefficiency was observed in Bchl-IgG compared to that of Bchl-Ser. This may be due to the specific binding between Bchl-IgG and protein A on the bacterium. Therefore, the results demonstrated the higher and specific efficacy of Bchl-IgG. In spite of its lower potency of binding, the preparation of Bchl-Ser was patented (EP0584552B1) [60]. Moreover, this molecule Bchl-Ser was further applied to study the photophysical behaviors, solubility in water, antivascular effect in solid melanoma tumors and tumor microenvironment [61,62,63].

### 3.3. Influence on Bacteriochlorophyll Aggregation

Bacteriochlorophyllides were used to study lamellar spacing in self-assembling aggregates. Chlorosomes contained bacteriochlorophylls surrounded by a galactolipid monolayer, which are responsible for harvesting of light in green bacteria. To study the role of esterifying alcohols of bacteriochlorophyllide *c* aggregation, different lengths of esterifying alcohols (C1, C4, C8 and C12) were conjugated with bacteriochlorophyllide *c* [64]. Such complexes were subjected to polar and nonpolar environments to study the aggregation behavior. Results showed that the hydrophobic interactions formed between longer esterifying alcohols in bacteriochlorophyllide *c* could drive the formation of bacteriochlorophyllide *c* dimers, while the shorter esterifying alcohol preferred monomers. The other study prepared different types of bacteriochlorophyllides (*c*, *d*, or *e*) to investigate lamellar spacing in self-assembling aggregates in chlorosomes [65]. Results indicated that scattering features of bacteriochlorophyllide c aggregates are similar to native chlorosomes. Self-assembling bacteriochlorophyll *c* aggregates are fully encoded in lamellar structures. In addition, bacteriochlorophyllides *c*, *d*, or *e* were trans-esterified with different alcohols to study the correlations between the length of esterifying alcohol and the spacing of lamellar spacing. Results showed that the lamellar spacing increased linearly in a length-dependent manner, indicating that lamellar spacing is proportional to the length of esterifying alcohol [65]. Taken together, the esterifying alcohols as geranylgeranyl, farnesyl and phytyl groups were observed in bacteriochlorophyllides. The variety of esterifying alcohols or lengths provide the diversity of bacteriochlorophyllides aggregates in chlorosomes. The esterifying alcohols may function as an anchor to lock bacteriochlorophyllides in the appropriate positions. Therefore, both of the types of bacteriochlorophyllides and esterifying alcohols in bacteriochlorophyllides affected the lamellar spacing in self-assembling aggregates.

### 3.4. For Dye-Sensitized Solar Cells Application

**Figure 6:**
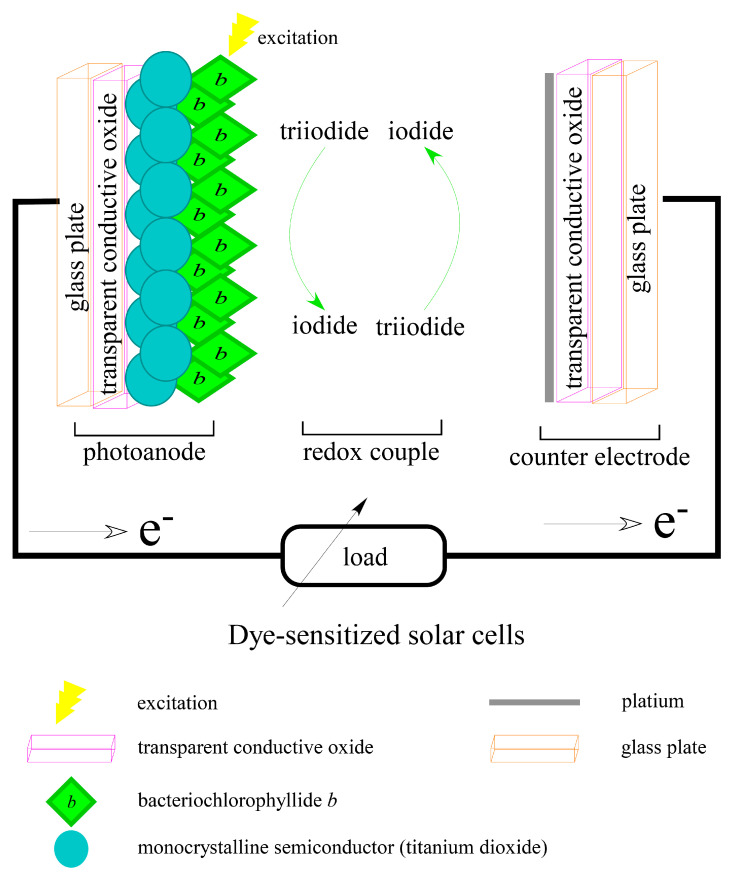
Schematic drawing of bacteriochlorophyllides used as a component of dye-sensitized solar cells (Modified from Shalini et al. [70]).

For applications in dye-sensitized solar cells, a patent (JP2014003938A) invented a new enzyme and method for producing bacteriochlorophyllides *b* or *g* [66]. The inventors described that bacteriochlorophyllides *b* or *g* have a longer wavelength shift than bacteriochlorophyllide *a*, which is beneficial to dye-sensitized solar cells [67,68]. Dye-sensitized solar cells are used to convert solar radiation into electrical energy. Such a device contained a transparent conducting oxide, dye sensitizer, a photo anode, electrolyte and a counter electrode. A photoanode was developed from mesoporous metal oxide layer, such as TiO_2_. Dye molecules as sensitizer anchoring on the surface of the photoanode absorb photos. A counter electrode was a glass coated with platinum. Electrons were released from excited dye molecules and finally migrated into external load circuit (Figure 6). Thus, by applying bacteriochlorophyllides *b* or *g* in dye-sensitized solar cells, this could increase the conversion efficiency of light energy and could extend the absorption wavelength to the near-infrared region [69,70]. Therefore, bacteriochlorophyllides in dye-sensitized solar cells provided a cost-effective alternative to traditional solar cells.

### 3.5. For Heme Synthesis

The synthetic substituted heme was produced by bacteriochlorophyll derivatives, due to the similar structure between heme and bacteriochlorophyllide. Generally, heme group and apomyoglobin generated a complexed named myoglobin. Wright and Boxer prepared zinc bacteriochlorophyllide *a* -apomyoglobin complexes as synthetic heme and determined the solution properties [71]. Apomyoglobin causes shifts and intensity changes of zinc-bacteriochlorophyllide *a*. The intensity of circular dichroism showed 3-6 folds increase in zinc bacteriochlorophyllide *a*-apomyoglobin. Another group (Marković et al.) investigated the stabilities between myoglobin, apomyoglobin and zinc-pheophorbide *a*, zinc-bacteriopheophorbide *a* by thermal and chemical denaturation [72]. Marković et al. found that the stability of zinc-pheophorbide-myoglobin is higher than zinc-bacteriopheophorbide-myoglobin [72]. The thermal unfolding temperature of zinc-pheophorbide *a* was 83.9 °C, while that of zinc-bacteriopheophorbide *a* was 82.6 °C. The recovery rate of zinc-pheophorbide was 92–98%, while that of zinc-bacteriopheophorbide was 40%. For the stability of synthetic heme complexes, native heme was the best, followed closely by zinc-pheophorbide-myoglobin, and zinc-bacteriopheophorbide-myoglobin. Therefore, bacteriochlorophyllides stabilize the apoprotein and the complex of synthetic substituted heme.

### 3.6. For Light Energy Harvesting Simulation

For studying the light energy system, a system for harvesting natural light energy and dissipation was designed as zinc-bacteriochlorophyllide dimers conjugated with four-helix bundle proteins [73]. To search suitable bundle proteins in this system, heme, heme-binding protein and its mutants were tested. The results indicated that zinc-bacteriochlorophyllide preferred to bind heme-binding protein, heme-binding protein mutants, and then heme itself. Zinc-bacteriochlorophyllide formed dimers within heme-binding protein and dramatically quenched the fluorescence of zinc-bacteriochlorophyllide. Therefore, this system could mimic the natural photosynthetic system to study the properties of light harvesting and dissipation [73].

### 3.7. Challenges and Future Perspectives

Bacteriochlorophyllides owned an intense electronic absorption in the red or near-infrared range; this offer was a more valuable and interesting opportunity in its applications. Although, it has been known that the feature of bacteriochlorophyllides is the reduced stability [39]. This reduced stability is due to the two reduced pyrrole rings in the structure of bacteriochlorophyllides. The problems of stability and high cost of production may lead to its delay in the functional study. In recent years, researchers have focused on the synthetic bacteriochlorophyllides derivatives that provided new opportunities for the enhancement of stability. However, there is still a growing demand and challenges on natural-occurring bacteriochlorophyllides due to the instability and production. To obtain a large amount of and purified natural bacteriochlorophyllides, we propose potential future perspectives:

a. New recombinant enzymes with high catalytic activity could be developed [10].
b. Extraction, isolation and purification of bacteriochlorophyllides could be optimized.
c. New preservation technology (nanotechnology) for bacteriochlorophyllides could be developed.

## 4. Conclusions

This review summarized the chemical structures and applications of bacteriochlorophyllides. The structural differences were described between the members and derivatives of bacteriochlorophyllide family. The source, correlation and synthesis route of all bacteriochlorophyllides were also clarified. Divinyl-chlorophyllide *a*, as an interaction hub, could be used to synthesize all bacteriochlorophyllides. The diverse applications of bacteriochlorophyllides included photosensitizers, immunosensors, lamellar spacing, dye-sensitized solar cell, synthetic substituted heme and light energy systems. To develop the extensive applications and gain access to the biological function of bacteriochlorophyllides, high purities of bacteriochlorophyllides are needed. This can be achieved by improving the purification strategies of bacteriochlorophyllides. Combined with advanced or cutting-edge technology, such as nanotechnology tools, the stability or bioavailability of bacteriochlorophyllides can be achieved. Accordingly, the potential application of bacteriochlorophyllide is increasing for extensive purposes. We suggest that the detailed mechanism of bacteriochlorophyllides in medical therapy, disease prevention, and health maintenance should for making bacteriochlorophyllides beneficial for human welfare.

## Figures and Tables

**Table 1:** The molecular formula, molecular weight and structure of bacteriochlorophyllides.

Name	Molecular Formula	Molecular Weight (g/mol)	Ring Structure	Structure
**B** **acteriochlorophyllide *a***	C_35_H_36_MgN_4_O_6_	633	Bacteriochlorin	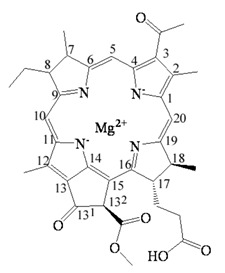
**B** **acteriochlorophyllide *b***	C_35_H_34_MgN_4_O_6_	631	Bacteriochlorin	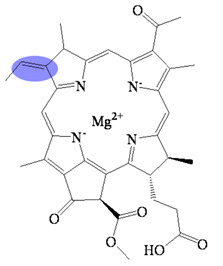
**B** **acteriochlorophyllide *c***	C_31_H_28_MgN_4_O_4_(R_1_)(R_2_)	559 (#)	Chlorin	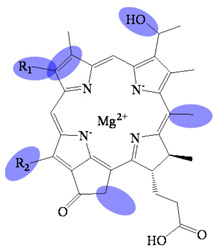
**B** **acteriochlorophyllide *d***	C_33_H_30_MgN_4_O_3_(R_1_)(R_2_)	545 (#)	Chlorin	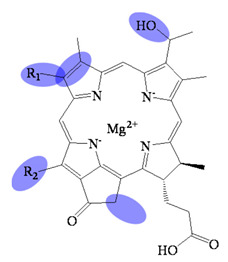
**B** **acteriochlorophyllide *e***	C_31_H_26_MgN_4_O_5_(R_1_)(R_2_)	572 (#)	Chlorin	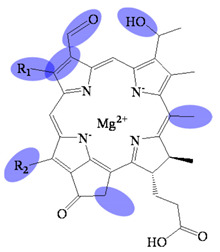
**B** **acteriochlorophyllide *f***	C_31_H_26_MgN_4_O_5_(R_1_)(R_2_)	559 (#)	Chlorin	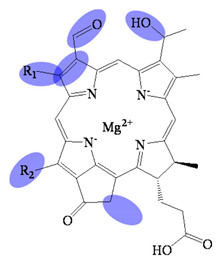
**B** **acteriochlorophyllide *g***	C_35_H_34_MgN_4_O_5_	615	Bacteriochlorin	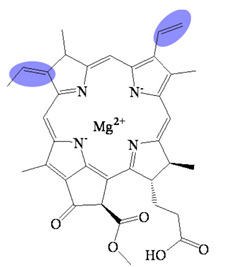

# Excluded of R_1_ or R_2_; R_1_ = ethyl, propyl, isobutyl or neopentyl; R_2_ = Methyl or ethyl; Note: The structure differences of bacteriochlorophyllide derivatives to bacteriochlorophyllide *a* are highlighted with spots.

## Data Availability

The study did not report any data, please exclude this statement.

## Sample Availability

Samples of the compounds are not available from the authors.

## Abbreviations

bacteriochlorophyllide-serine (Bchl-Ser); bacteriochlorophyllide-Immunoglobulin G (Bchl-IgG); WST-11 and dextran (WST-D).
